# Household food waste from a settlement perspective in Cape Town South Africa

**DOI:** 10.1038/s41598-025-26239-y

**Published:** 2026-03-03

**Authors:** S’celo Ezra Madondo, Elaine Sinden, Catherina Schenck

**Affiliations:** 1 Institute for Social Development, University of the Western Cape, Robert M. Sobukwe Rd. Bellville 7535, Cape Town, W/Cape RSA South Africa; 2 Centre of Excellence in Food Security, University of the Western Cape, Cape Town, Robert M. Sobukwe Rd. Bellville 7535, W/Cape RSA South Africa; 3 DSI/NRF/CSIR SARChI, Chair in Waste and Society, University of the Western Cape, Cape Town, W/Cape RSA South Africa

**Keywords:** Consumers, Food security, Food waste, Household, Ubuntu (Botho), Environmental impact, Sustainability, Environmental sciences, Environmental social sciences

## Abstract

Food security, greenhouse gas emissions from the food supply chain, and waste disposal are three of the most pressing global challenges. Food waste impedes food security, environmental sustainability, and economic resilience, especially in rapidly urbanizing destitute settlements of the Global South, where structural vulnerabilities intensify the issue. This study examines the extent, nature, and drivers of household food waste in Wallacedene, a less privileged settlement on Cape Town’s periphery. Using a mixed-methods approach, we conducted 85 household surveys alongside a focus group discussion to explore food waste practices, perceptions, and challenges. Findings indicate that 85% of households discard edible food, predominantly vegetables, bread, and fruit. Key drivers include inadequate meal planning, restricted access to refrigeration and storage facilities, irregular income affecting purchasing habits, and limited awareness of food preservation techniques. While many participants reported guilt, sadness, and frustration over food waste, their ability to reduce it was constrained by structural and material barriers. This study underscores the intricate relationship between individual behaviors and systemic limitations, arguing that household food waste requires a multifaceted approach beyond behavior change campaigns. Effective interventions must integrate education, infrastructure improvements, and community-driven solutions adapted to local contexts. Additionally, households should align food disposal decisions with ethical and moral principles rooted in Ubuntu (Botho), reinforcing collective responsibility and minimizing waste through economic and socially inclusive food-sharing practices. These findings provide empirical evidence for food waste in low-income African settlements, offering actionable insights for policies supporting Sustainable Development Goal 12.3 of the United Nations.

## Introduction

Food waste is increasingly recognised as a critical global issue, with substantial implications for food security, environmental sustainability, and economic resilience^1–3^. The Food and Agriculture Organization (FAO) estimates that approximately one-third of all food produced globally is lost or wasted, contributing significantly to greenhouse gas emissions, pressure on water and land resources, and the ethical dilemma of disposing edible food amid persistent hunger^4^. Household-level food waste is particularly concerning, as it directly undermines Sustainable Development Goal (SDG) 12.3 of United Nations^5^. The Economic Research Service (ERS) of United States of America’s Department of Agriculture (USDA) defines food loss as follows:

*“The amount of food available for human consumption – after removing bones*,* pits*,* peels*,* and other nonedible parts – that is not consumed for any reason. It includes moisture loss and cooking shrinkage; loss from mold*,* pests*,* or inadequate climate control; and food waste. Food waste is a subcomponent of food loss*,* and examples include edible food discarded by retailers due to color or appearance and plate waste thrown away by consumers.”*^6^.

Food Loss and Food Waste (FLW) in this study, refer to the disposal or non-food use of safe, nutritious food, as well as losses occurring between production, markets, and human consumption. Stancu, Haugaard and Lähteenmäki^7^ together with Stuart^8^, all define food wastage as encompassing both FLW categories. FLW has a triple adverse impact: (a) the waste of resources – including water, energy, and labor – utilized in production and distribution; (b) the socioeconomic repercussions of food insecurity; and (c) environmental consequences, including greenhouse gas emissions from food production, processing, and disposal^9,10^. The challenge lies in sustainably meeting the world’s escalating food demand while ensuring food security, an issue explored in numerous studies^11^. While, the evolving definition of food security now emphasizes sustainability as its fifth pillar^12^.

According to projections, the global population will reach 9.6 billion by 2050^13^. The United Nations’ SDGs highlight that over 10% of the world population live in extreme poverty, with the majority residing in Sub-Saharan Africa (SSA)^5^. FLW contributes to approximately 26.8% of global hunger, posing a severe challenge in SSA^4^. Beyond its direct impact on human livelihood, food waste has broad social, economic, environmental, and climate consequences^14,15^. Humanity may require the equivalent of three planets’ resources to sustain the population by 2050^16^. With one-third to a half of global food production wasted^2^, discarding food is both unethical and unsustainable, particularly when millions face hunger. Indigenous knowledge and ethical frameworks such as Ubuntu (Botho)^3,17–23^, underscore a need for economic and socially inclusive solutions to FLW.

South Africa is estimated to lose 10.3 million tons a year of edible food earmarked for human consumption, and this is comparable to 34% of local food production^24,25^. Food waste is more troubling in a context where food insecurity persists, across both rural and urban poor populations^26^. Research by Battersby and Peyton^27^ has shown that in low-income urban areas such as Cape Town, food access is shaped by geography, income volatility, and inadequate infrastructure, making food waste a paradoxical yet growing problem. Informal settlements, in particular, face compounded risks due to irregular food access, limited refrigeration, and constrained waste disposal systems^11,28^. Also, the understanding of cultural practices on the impact of food waste cannot be ignored, according to Pasha et al.^22^, as these practices have a major influence on food waste. Culture, in the lives of South Africans is deeply embedded^22^. Values, principles and ways of life thus have an impact on natural resources like water.

Issues of water scarcity intensify the consequences of FLW in context of SA. Approximately 22% of the country’s crop production water is lost annually due to food wastage^10^. This limits the agricultural sector and given South Africa’s status as the 30th driest country in the world, these losses are not only environmentally unsustainable but also economically and socially costly. Similar patterns have been observed across Sub-Saharan Africa, where studies have increasingly linked household food waste to infrastructural deficits, food literacy gaps, and socio-cultural norms, inter alia^1,29^.

Despite global devotion to FLW by numerous scholars such as Schott and Andersson^30^, Thyberg and Tonjes^15^, and many others, together with growing national attention to the challenge, empirical evidence from informal poor urban settlements in Sub-Saharan Africa remains sparse. Similarly, as noted by Madondo^3^, Oelofse, Muswema and Ramukhwatho^28^, there is limited data on household-level food wastage in South Africa, particularly in areas where waste is often unmeasured and disposal practices are informal and not monitored. Existing studies tend to focus on post-harvest and supply chain losses, with relatively little attention to consumption-phase losses in domestic settings^31,32^. This study addresses this research gap by examining the scale, nature, and drivers of household food waste in Wallacedene, an informal settlement located in Cape Town, South Africa.

## Background and area context

### Research background

Added to raising food insecurity, food wastage poses significant ecological, economic, and social challenges, contributing to greenhouse gas emissions, depletion of natural resources, and water eutrophication^33,34^. As sustainability initiatives gain traction, the recognition of food waste as a global crisis has intensified. The United Nations Millennium Declaration (2000) initially sought to address food waste but lacked adequate support, yielding limited impact^14^. However, at the UN General Assembly session in 2015, 193-member states, with the participation of civil society, adopted an agenda endorsing 17 Sustainable Development Goals (SDGs)^5^. These goals aim to eradicate poverty by 2030 while fostering environmental protection and human well-being. Specifically, Goal 12 emphasizes sustainable consumption and production patterns, including a commitment to halving global food waste per capita at the retail and consumer levels and reducing losses throughout supply chains by 2030^35^.

Beyond environmental concerns, food wastage intersects with food security, poverty reduction, and economic stability. The SDGs advocate for responsible resource use, ensuring sufficient food supply for current and future generations, projected to reach nine billion by 2050^36^. Economic sustainability hinges on both environmental conservation and societal welfare. In South Africa, food rescue initiatives play a crucial role in waste reduction and hunger alleviation. The Warehouse Food Banking (WFB) model used by various associate organisations locally and beyond involves sourcing, collecting, and storing edible surplus food from farmers, manufacturers, and retailers, with a focus to rescue the food and to feed vulnerable communities by redistributing it to vetted beneficiary organizations^37^. The Food Bank South Africa (FBSA) network of organisations provides a platform for food donations from producers, manufacturers, retailers, government agencies, and individuals. In 2016 alone, FBSA distributed 3,348 tons of food, equating to over 11 million meals – food that would otherwise have ended up in landfills^11^. Key players in charity here are FBSA, SA Harvest and Food Forward SA, however, logistical and infrastructural barriers limit their outreach in densely populated informal areas. While, household-level data, particularly within poor and informal settlements remains rare. This lack of data hampers the development of targeted strategies suited to the realities of low-income communities. Addressing this research gap thus calls for a better understanding of these localities.

### Contextualising the study area

Wallacedene started in the spirit of Ubuntu (Botho), as an informal housing settlement located in the northeastern suburb of Cape Town^3^. This African way of life as espoused by multiple scholars^3,17–23^ was noted late during the 1980 s in South Africa, as relaxation of pass laws allowed rural populations to migrate more readily to urban centres. Groups of homeless people came and settled communally as the first informal inhabitants of an abandoned Kraaifontein farm, Uitkyk, and gradually established themselves as a community^38^. Areas such as these are commonly known for joblessness, hunger, poverty, lack of hygiene and related issues impacting the fabric of the community’s wellbeing through crime and deterioration of social and cultural values^38,39^.

**Fig. 1:**
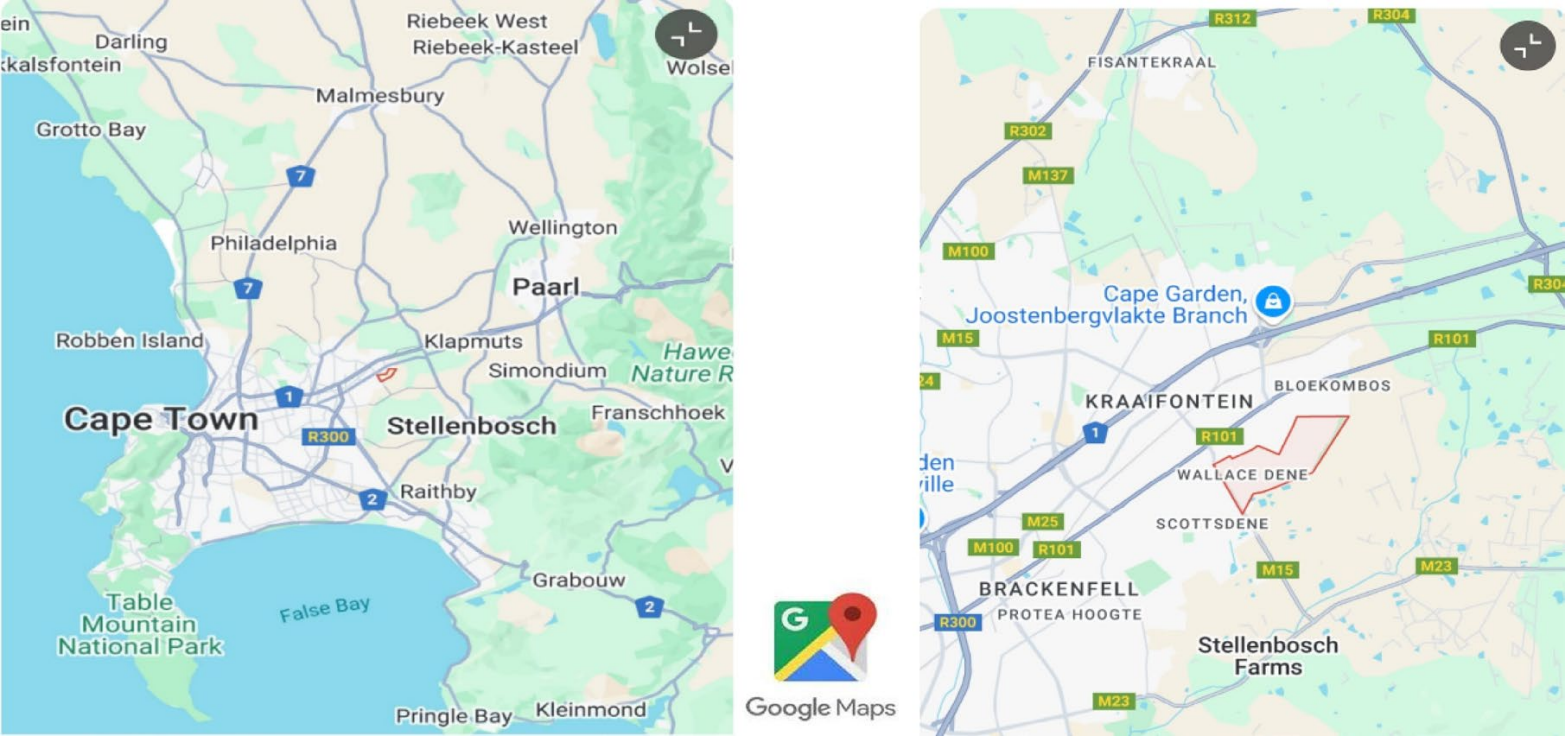
Geographical location of Wallacedene in Kraaifontein, CT. Zoom close-up level map. Source: Google Maps (2021)

Historical foundations of Wallacedene from its ground-breaking Western Cape High Court (WCHC) decision 6826/99 of the Grootboom case, changed the future of spatial planning in the region and the country^40^. This case was highly momentous regarding food security, eviction matters and social welfare in South Africa. Davis^40^ ordered the City of Cape Town and others “to provide adequate and sufficient nutrition, shelter, health and care services and social services to all of the applicants and children”, instead of enforcing an eviction. Geographical maps in Fig. 1 locate the study area about 14 km from Bellville, the nearest town^41^. Wallacedene occupies a locality radius of 0.60 km2 of land space and is situated 30 km in the northeast periphery of CT, adjacent to the Old Paarl Road and N1 route-north^42^.

Statistics SA (2011) says during 2004 Wallacedene had an estimated population of 21,000 people, the majority of whom were Black, followed by Coloured people. The area population grew significantly from 21,000 in 2004 to 36,583 by 2015, all housed in over 10,000 households^26,38^. The City Population demographic report of 2011 recorded an equal gender distribution in Kraaifontein^42^. While the official household size averaged 3.5 people^43^, estimates by the City of Cape Town (CCT) suggested 4 to 5, with independent assessments indicating densities of 6 to 8 per household [63].

High population density leads to severe overcrowding, straining essential services such as water, sanitation, and waste management [63]. In South Africa’s urban areas, household waste collection typically follows the RSA National Domestic Waste Collection Standards^10^. However, rising population pressures exacerbate challenges in waste collection, infrastructure, housing shortages, and informal settlement advance. These demographic realities, beyond food security, underscore the need for integrated urban planning to address service delivery and community development in such areas.

## Research materials and methodology

### Study design

A mixed-methods approach was adopted in the study, integrating qualitative and quantitative research methods to investigate household food waste in Wallacedene, South Africa. A case study process was used, incorporating a qualitative design with elements of quantitative analysis to provide a well-rounded perspective on food waste management. Secondary data collection utilised documents, reports, a WCHC decision 6826/99 of the Grootboom case^40^, as well as various municipal solid waste management data in the City of Cape Town, Western Cape Government, and South Africa. The CSIR and other scholars estimate that only 13 out of 284 municipalities across South Africa have some form of data on food waste^25,44^. Questionnaire surveys were then also used to collect structured quantitative and qualitative data so as to eliciting descriptive feedback from respondents. With this, a Focus Group Discussion (FGD) was held to explore participants’ experiences and behaviors in-depth.

### Sampling

Purposive sampling technique targeted household high incidences of waste dumping sites in Wallacedene. The sample size included 100 households, with 85 questionnaire survey respondents. A random sample of 9 participants from the survey pool was selected for the FGD. However, due to logistical constraints, only 7 participants attended. The study also focused on participants who had to meet specific criteria, including that of individuals who had lived in the area before the construction of government-subsidized Reconstruction and Development Programme (RDP) houses. Moreover, due to a mixture of languages, respondents capable of articulating experiences in English regarding household food-handling behaviors were prioritised.

### Data collection

Data was gathered through multiple sources as described, firstly from a review of secondary data. Then, from 100 distributed questionnaires there were 85 completed responses. The questionnaire included both closed-ended and open-ended questions to capture quantitative and qualitative insights into food waste types, frequency, amounts, and disposal practices. Added, a small FGD with 7 participants was held to explore household food waste behavior in greater depth. The FGD was audio-recorded using two smartphones, with written notes taken for further validation.

### Data analysis

A mixed-methods approach combined statistical and thematic techniques. Quantitative data analysis was processed using Microsoft Office Excel, presented through graphs, charts, and tables to describe demographics of study subjects and food waste patterns. For qualitative analysis, FGD were transcribed, interpreted and coded into themes and patterns aligned with research objectives. Consideration was given to methodological biases, particularly self-reporting limitations as highlighted by Giordano et al.^1^, which emphasise discrepancies between stated behaviors and actual food waste disposal habits.

### Limitations and study contribution

The study acknowledged challenges, including a limited sample size due to resources constraints and logistical issues preventing 2 of the 9 invited FGD participants from attending. All other practical difficulties like technological connectivity and capturing of data, were all circumvented leading to the collection of valuable qualitative data. Beyond collecting data and analysing food waste behavior, this study also raised awareness at multiple levels, including households, municipal policymakers, educational institutions, and political circles. The findings contribute to academic knowledge by addressing food loss and waste (FLW) research. The study and experimental protocols were approved by the Humanities and Social Science Research Ethics Committee of the University of the Western Cape (UWC) in South Africa, and all methods were performed in accordance with the relevant guidelines and regulations. Only adults were interviewed for this study and informed consent was obtained from all participants.

## Study results and discussion

**Table 1:** Proportional representation of food waste generation and cost in SA.

Produce	% Waste	% Cost
Fruit and vegetables	44	36
Meat	7	28
Cereals	26	7
Roots and tubers	9	5
Fish and seafood	2	13
Milk	8	6
Oil seeds and pulses	4	5
Commodity Food Groups	100	100
	Total %	

Source: Oelofse (2015).

According to Oelofse and Nahman^20^, the annual cost of food waste in South Africa is estimated to be R2,012 billion and national data in Table 1 for FLW is measured in a thousand tons per year, with its cost measured in billions per year. The backdrop in Table 1 foregrounds all subsequent study data from Wallacedene, highlighting the significant economic impact of food waste and underscoring the need for effective strategies to mitigate FLW at the household level. Economic implications of food waste are intricately linked to the market prices that govern the food value chain^20^. These prices encompass the various stages of the food value chain, from agricultural production to consumption, as illustrated. By exploring the patterns and practices of food waste among households, this research presents an opportunity to fill a crucial gap, aiming to contribute to a more comprehensive understanding of FLW since there is a lack of sufficient subject data in South Africa^3,11,25,28^. When addressing scarcity of information on household food waste in South Africa^3,28^, it is crucial to juxtapose the deficiency with impact of FLW to the economy. This situation underscores the need for targeted interventions, and for this reason, focus is on household food waste to align with ethical and moral principles of Ubuntu (Botho)^3,17–23^, together with Sustainable Development Goal (SDG), target 12.3^5,35^. So, the subsequent sections of this study present key findings on household food waste, as reported by study participant in Wallacedene, shedding light on the complexities of food waste at the household level in the area.

### Prevalence of household food waste

**Fig. 2:**
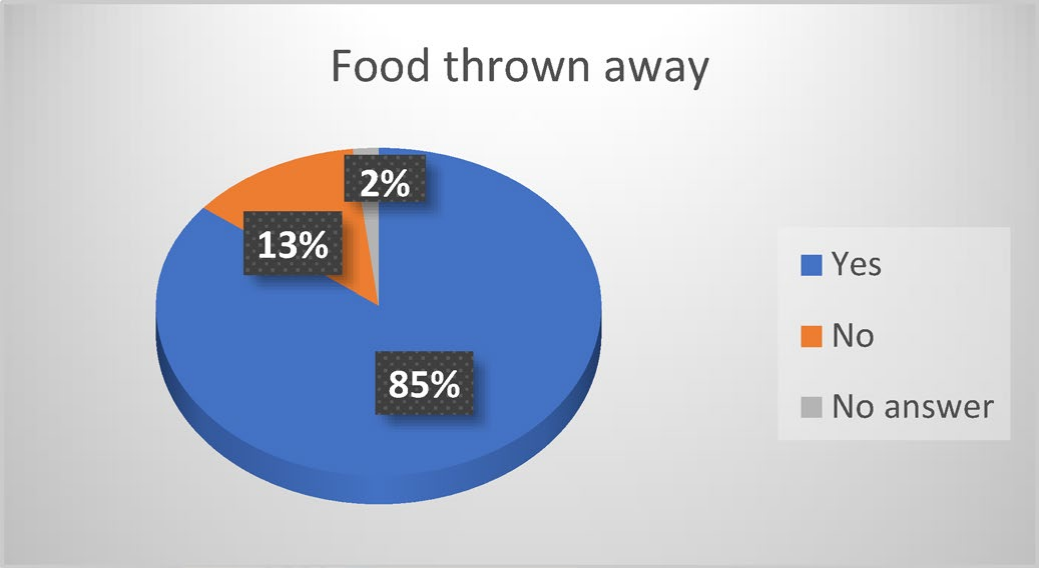
Residents who agree they throw food away. Source: Research data, 2021.

A significant proportion of participants in Fig. 2 (85%, *n* = 72 out of 85) reported discarding edible food (‘n’ refers to the number of participants or sample size that corresponds to the percentage mentioned). This confirms findings from other South African urban settlements, where household-level food waste is driven by poor food management skills and limited awareness of food insecurity^11,28^. The study participants identified several contributing factors: inadequate meal planning, over-purchasing, food spoilage due to load shedding (interrupted electricity supply), and limited use of leftovers. These drivers align with studies in both the Global North and Global South that attribute household food waste to behavioral and infrastructural constraints^1,33^. From the perspective of the Theory of Planned Behavior (TPB)^39^ many participants showed a positive attitude toward reducing waste (e.g., expressing guilt or concern) but faced barriers related to perceived behavioral control, such as lack of refrigeration or unpredictable electricity. This gap between intention and action, also known as the intention – behavior gap, has been documented in prior food waste literature^32^.

### Most commonly wasted food types

**Fig. 3:**
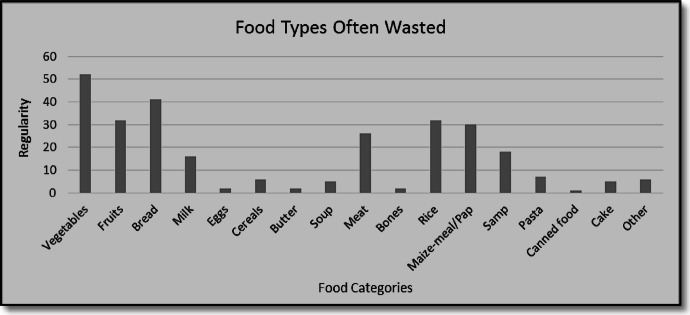
Food types often wasted. Source: Research data, 2021.

Figure 3 displays recurrence in food category types wasted, as items are measured in repeat mentions numbered from participants responses reflected on the regularity Y axis. Vegetables account for the highest proportion of food waste (18.4%), followed by bread (14.5%), fruit (11.3%), and rice (11.3%). Other frequently wasted items included mealie meal (used to prepare pap) at 10.6%, and various meats at 9.2%. These results reflect findings by Katajajuuri et al.^33^ together with Oelofse and Nahman^25^, which indicate that perishables, especially fresh produce and bread are most vulnerable to waste. Meat waste is particularly noteworthy due to its environmental footprint and higher monetary cost. Cultural consumption patterns also influenced these results^22^. For example, the relatively high frequency of discarded steam bread or braai meat leftovers, suggests that socio-cultural food preferences and serving customs play a role in over-preparation and waste. However, these must be interpreted cautiously and grounded in broader household dynamics rather than cultural generalizations.

### Disposal practices

**Fig. 4:**
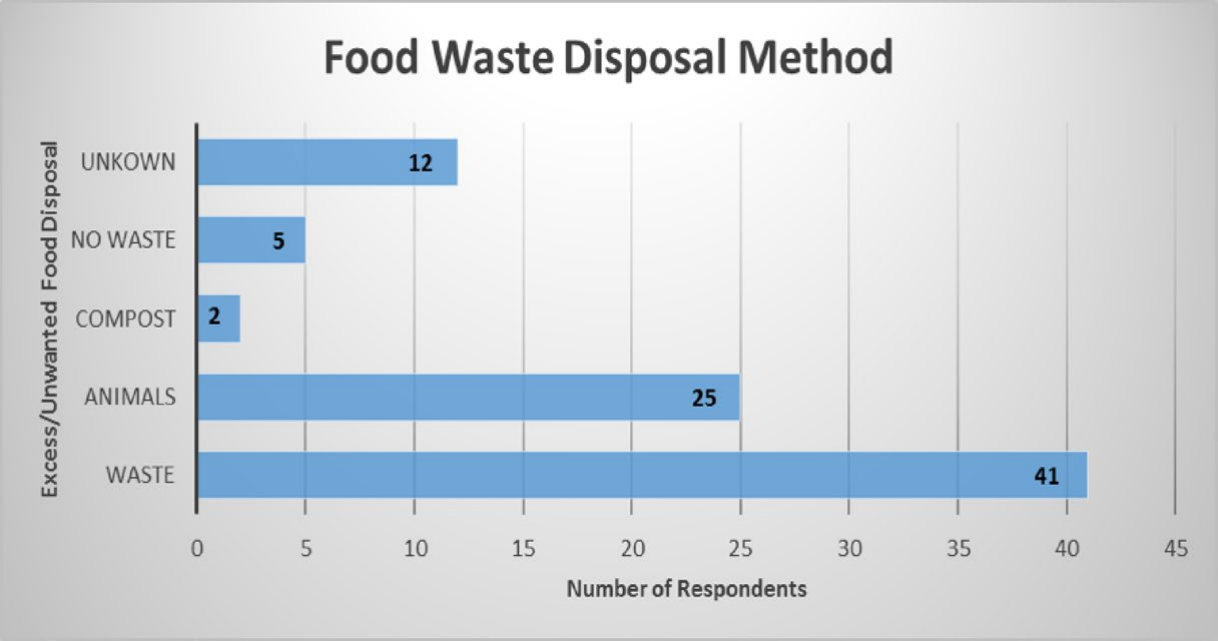
Food waste disposal method. Source: Research data, 2021.

In this Fig. 4 chart, plurality of participants in the study (48.2%, *n* = 41) reported discarding leftover food in municipal waste bins. Others disposed of food through informal means such as open dumping, sewer flushing, or composting. Only a small number (5.9%, *n* = 5) reported regularly offering food to the needy or using leftovers as animal feed. These findings are concerning in light of South Africa’s limited landfill capacity and challenges in municipal solid waste management^24^. The improper disposal of food waste not only exacerbates environmental degradation but also contributes to blocked drainage systems and public health risks in informal settlements^45^.

### Quantities and frequency of waste

#### Food waste quantities

**Fig. 5:**
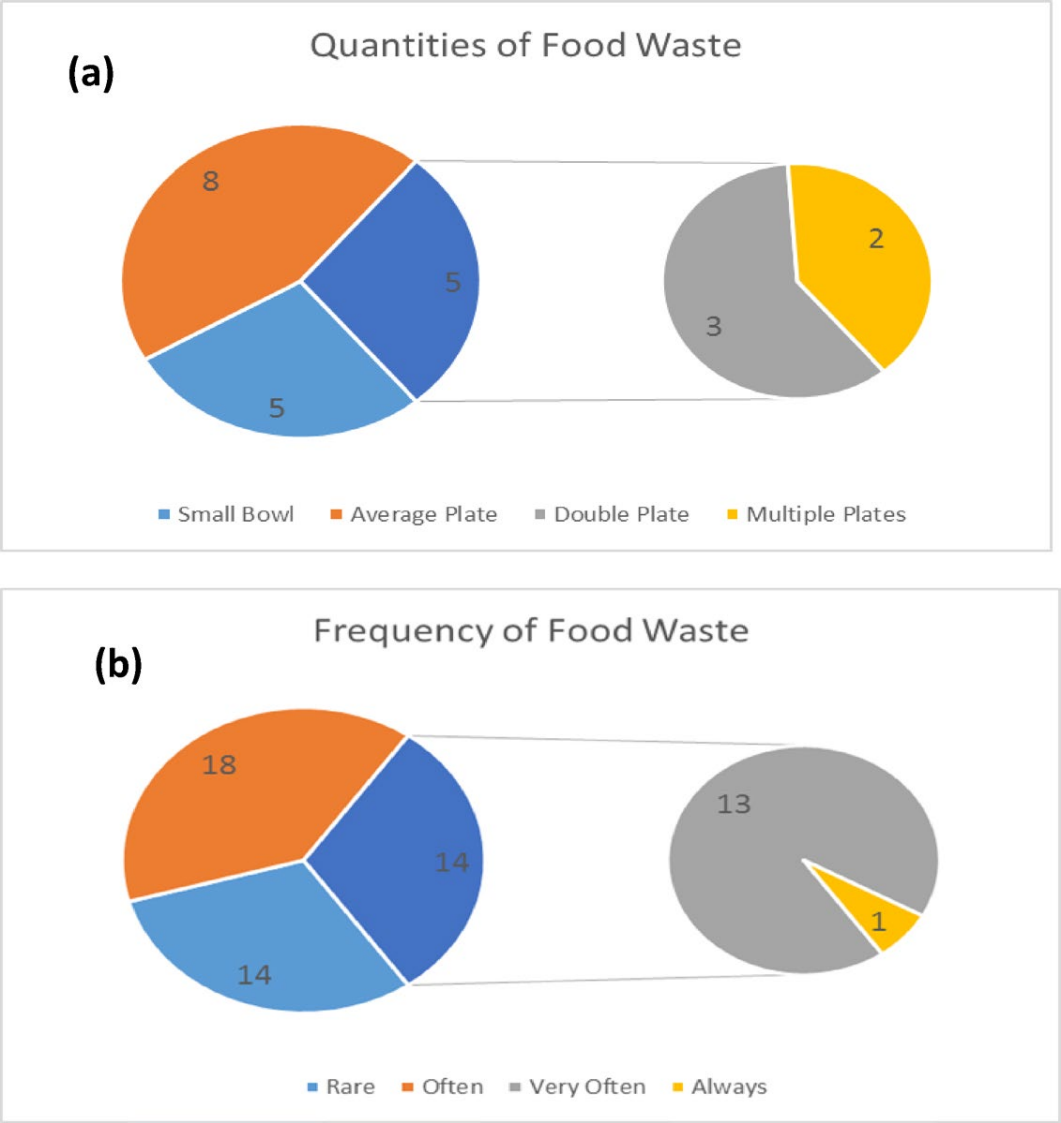
Quantities of food waste. Source: Research data, 2021.

From (43.90%, *n* = 41) respondents who alluded to mindful food waste, Fig. 5 shows 5 respondents (5.88%, *n* = 41) who reported regular food waste in a small bowl, while 8 of them (9.41%, *n* = 41) indicated that one standard size plate of food normally goes to waste. Whereas, 3 respondents (3.53%, *n* = 41) alluded to double standard size plates and 2 respondents (2.35%, *n* = 41) reported multiple plates of edible and nutritious food being wasted. The numerical values observed in food waste within bowls and plates for this category may not be directly comparable to findings from other studies that quantify FLW in metric tons. Caution should be exercised hence, when drawing comparisons between micro-level household waste assessments and broader quantitative FLW estimates. Even though food waste quantities were generally modest in absolute terms, these were significant relative to household affordability, since Wallacedene is a low-income area.

#### Frequency of food waste

Participants typically reported wasting the equivalent of a small bowl or plate of food, but such portions were repeated regularly. In terms of frequency: Fig. 5 section shows 14 respondents (16.47%, *n* = 85) saying it is ***rare*** that their food is wasted. Automatically, here is a possible opportunity for a cohort of households who may speedily improve their food handling behaviour. On another side, a vast number of 18 respondents (21.18%, *n* = 85) granted that they waste food ***often***, while 13 others (15.29%, *n* = 85) admitted that they waste food ***very often***, as 1 respondent (1.18%, *n* = 85) said in their household, food is ***always*** wasted. These frequencies suggest that even small, recurring waste volumes may aggregate into substantial losses over time. As food prices rise, the economic impact of such waste becomes increasingly burdensome, especially for low-income households with limited food budgets^26^.

### Emotional and attitudinal responses

**Fig. 6:**
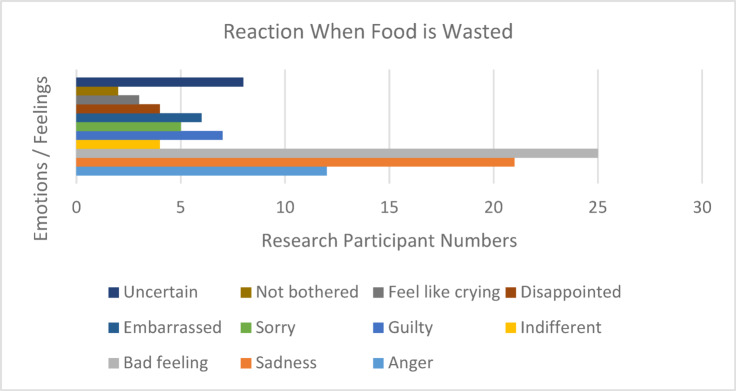
Participants’ feelings when edible food is thrown away. Source: Research data, 2021.

Figure 6 illustrates 11 emotion types expressed by respondents when food is wasted, totaling 97 occurrences in the entire study. This figure is separate from the study sample size and reflects a list of feelings expressed and their frequency. During the analysis it was clear that in a recurrent feeling of 25 times (25.77%, *n* = 97), respondents expressed a bad feeling, 21 times (21.65%, *n* = 97) some said they felt sadness, 12 times in the study (12.37%) others associated their feelings with anger, 8 times (8.25%, *n* = 97) some said they were uncertain, 7 times (7.22%, *n* = 97) some felt guilty, 6 times (6.19%, *n* = 97) others linked their feelings with embarrassment, 5 times (5.15%, *n* = 97) others felt sorry while 4 times (4.12%, *n* = 97) a few stated feeling indifferent and 4 times again (4.12%, *n* = 97) some felt disappointed, while 3 times (3.09%, *n* = 97) some said they felt like crying, and only on 2 occasions (2.06, = 41) did respondents say they were not bothered. The latter indicator of 2 citations, showing a nonchalant feeling, confirms, as stated earlier that almost all respondents are very much conscious and often intentional of their food waste conduct. So, based on the research findings, it is evident that even individuals who exhibited indifference (4 respondents) or uncertainty (8 respondents) regarding food waste, could enhance their habits if prompted with access to full and well-structured information.

These results reinforce that moral and emotional norms shape food waste behavior^22^. According to the TPB, emotions and social norms are key predictors of behavioral intention^39^. However, the inability to act in line with these norms, due to structural limitations highlights the importance of addressing both infrastructural and educational gaps. Moreover, there is a lack of social responsibility among participants, which is rather divergent to Ubuntu (Botho) local values^3,17–23^, and proper food and nutrition behaviour. With regards to nature resources, including food, the African view of morality and ethics is based on a contextual detail of ‘I am because we are’^3,21^. Its focus is on harmony in that membership in the community is, by and large, sufficient to be entrusted with an adequate portion of its land or other major kinds of wealth (traditionally cattle), supposing one continues to make good use of it and does not let it go to waste^18,19^. In traditional SeTswana society – and indeed across the Nguni people and all native tribes of South Africa and many related cultures of Southern Africa – it would be considered theft if one slaughtered an animal and kept the meat for oneself or gave it only to one’s nucleus family^18,19^.

According to Russell et al.^32^ and Stancu et al.^7^, responses such as the above emotions and feelings show that direct efforts to change consumer attitudes and behaviour towards food waste can potentially lead to a decline in its linked behaviour through intentional processes. Single-worded types of moods and such kinds of emotions as articulated by respondents in this study to describe how they feel when edible and wholesome food is thrown away, are a testimony to a plausible future scenario when community members are afforded food waste education and food security knowledge. After all, Williams et al.^46^ confirm for example that in general, about 50% and more of household and consumer food waste occurs due to the food not being used in time. Feeling a sense of guilt on the other hand as some participants indicated, appears prominently in Russell et al.^32^. Among study subjects, three declared this sense in dual wording, e.g., ‘Heartbroken and angry’ or ‘Heartbroken and sad’, with one strangely saying, ‘Good but sad’. All are placed under ‘Feel like crying’. Here is a very emotional response illustrating some financial anxiety from Participant 2 in the Focus Group Discussion (FGD):

I wanted to say, I feel bad when I see food thrown away. This is because, when you think as a breadwinner, looking there at that food and seeing your money. Or seeing these carefree kids [irresponsible children] and you then think, “No man, this is a real waste” [of food]. When you start questioning and talking about the waste, then you hear them say you are grumpy about food, when deep down, you know this difficult thing of working. You work hard for it [money], you know? You have worked so hard for this and these people [kids] cannot see this. For example, now we even have lost Ubuntu (Botho), our humanity to receive visitors. We have become unwelcoming to extended relatives [family] and strangers. We cannot even allow our families’ child [sister/brother] to come visit from the rural areas.

Another participant bared her inner emotion when food is thrown out in the open, thus polluting the environment. Participant 3 (FGD) expressed herself thus:

I think of the environmental impacts, as it makes our open spaces filthy. In Wallacedene it is embarrassing and a shame how our spaces are so full of rubbish that includes food items. Everywhere one goes, we find discarded items that contain food and other organic material; it’s a shameful feeling.

As the focus group discussion progressed, other participants also reacted to the emotional dialogue with one sharing a passionate interaction with her 9-year-old son, Participant 1 (FGD) said:

You see, it hurts me. My son, Miso is not an eater. He is a light eater – if there is anything like that. Every time I give him food, I make sure he has his plate. He wants to choose, saying, “Mommy, I want bread with cheese”… while I have prepared a full meal and everything. Yesterday, I was telling him, “My boy, it pains me that your plate of food, to another child who has no food, this meal will mean everything. This food that you have to eat, is in a high-class category-meal, and in your eyes, it means nothing or is less valuable”. So now, I take it that he does not understand since he is aged 9 and still young. I mean to say, you know me if I were to see a piece of a chicken wing, some meat on my plate, and when I offer it to you and you hold it and say, “I don’t want this meat”, it breaks my heart.

Finally, a further sensitive response came from another FGD participant, who observed:

So, it hurts and is painful honestly, to see a full pot of food thrown out by someone. For example, a full pot of rice is thrown out there. The very person would then come back and wash the pot and scoop another big portion of fresh rice from the container and pour it into the pot to prepare it for cooking on the stove. Then you ask yourself, “Haaibo! What is this?” Just today during the day, I was saying to someone, “You know, I feel such pain because we used to have stacks of meat that you found, placed there”. I would come from work and find chicken breasts and meat pieces lying there in the kitchen area and someone would tell me they do not eat *umkhwephe* (chicken breasts). You would then ask yourself, “Does this person know how hard I have worked for us to have this meat?” So, this thing of food waste hurts and gives me pain, honestly. I feel as if that is the reason. Hence, I say to them, I tell them at times, “If you guys knew where I originally grew up, I never had a proper chance to eat nourishing and nutritious food.” Participant 2 (FGD).

All this vastly qualitative data as presented and discussed, is a step ahead in resolving challenges of information gaps for far-reaching trials and data scarcity regarding consumer feelings on food waste, particularly in communities across many developing economies of the Global South, as Russell et al.^32^ and Gustavsson et al.^14^ state. However, this study had limitations in its subject investigation still, such as a great deal of food waste related issues pertaining to solid waste and municipal waste collection that could not be addressed. This included the broader municipal management of the dumping sites of waste at various public open spaces across Wallacedene and at landfill sites. Also, the study was conducted fundamentally for partial fulfilment of academic requirements, hence the limited sample size. Moreover, for practical and logistical reasons, primary sources from which data was collected, using questionnaires and FGD feedback focused specifically on targeted Wallacedene settlement areas. While, some participants may have observed/reported on other people (e.g., their children, neighbours or friends) wasting food, not themselves. Such information could not be filtered and their reports were recorded as given. Research data was statistically and thematically analysed.

## Recommendations to curb household food waste

### At the household level

Many households in Wallacedene lack adequate infrastructure, food literacy, and storage capacity. The following interventions are recommended: (i) *Food planning education* - Public education campaigns and community workshops can promote meal planning, use of shopping lists, and appropriate portion sizes. Studies show these practices can significantly reduce domestic food waste^25,47^; (ii) *Safe storage practices* - Awareness about how to safely store perishable foods, without or with limited refrigeration can improve food longevity. Low-cost solutions such as cool boxes or shared community refrigeration (where feasible) may be piloted; (iii) *Food reuse guidance* - Techniques for repurposing leftovers can be shared via radio, social media, and local NGOs to reinforce sustainable habits.

### At the school and community level

Schools and community centers are important platforms for shaping long-term behavior. Curriculum integration i.e. integrating food security and waste education into school programs can promote early awareness and behavioral change, according to Papargyropoulou et al.^48^. In addition, community awareness campaigns, such as local events, poster campaigns, and interactive sessions can demystify food labeling (e.g., “sell by” vs. “use by”) and correct misconceptions about edible food waste^7,47^. As Williams et al.^48^ highlight, the importance of clear labeling and packaging in reducing food waste, should be a consumers’ priority.

### At the government and policy level

**Fig. 7:**
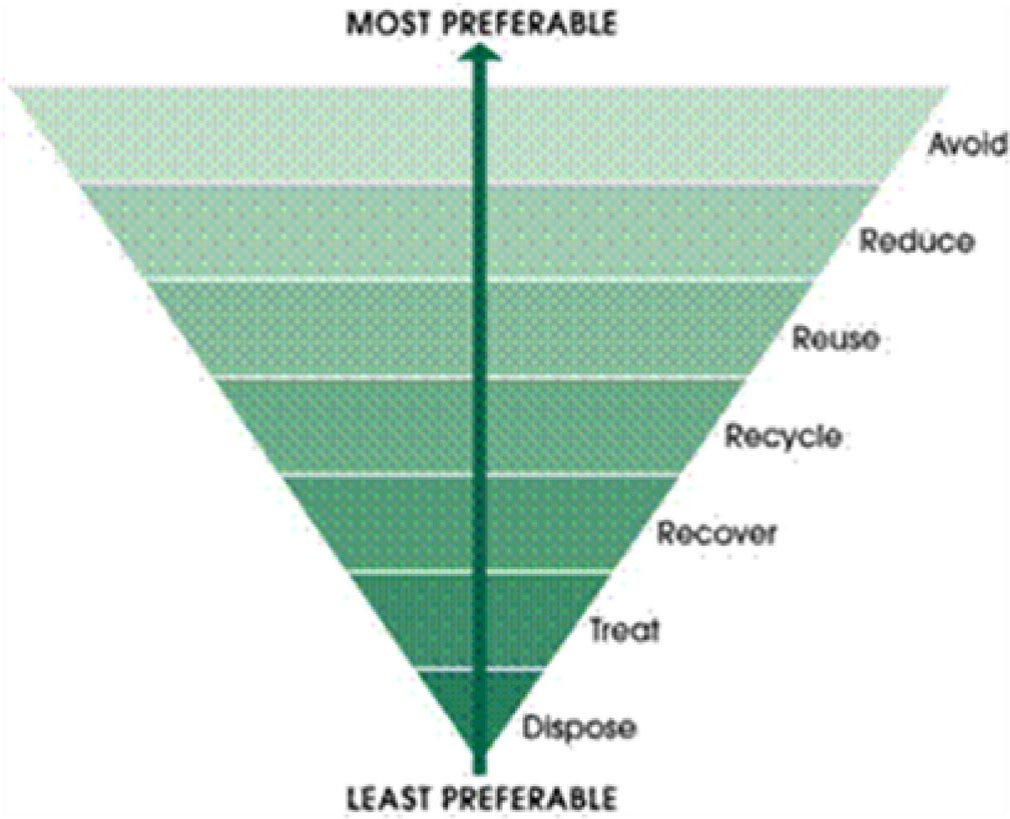
Preferable waste handling hierarchy. Source: Green Industries, S.A. (2020)^49^.

Local and national authorities play a critical role in enabling behavior change through policy and service provision: (i) *Infrastructure development* - Improved and consistent waste collection in informal settlements, combined with separation at source, can support food waste diversion to composting or animal feed; (ii) *Support for food redistribution* - Strengthening partnerships with food recovery organisations such as SA Harvest and Food Forward SA, can help redirect surplus edible food to vulnerable communities. However, logistical barriers in informal areas must be addressed; (iii) *Policy incentives* - Municipal and provincial governments can offer incentives (e.g., tax deductions or recognition awards) to retailers and hospitality businesses that donate unsold but edible food. Also, various opportunities are recently proven to accrue economically from leveraging food waste, beyond food security initiatives^10^. Governments, in particular municipalities and other relevant state entities must thus prioritise the exploration of job opportunities and sourcing commercial items within the food waste economy. This should be in line with e.g. preferable waste handling methods as outlined in Fig. 7.

### At the business and retail sector

Businesses have the potential to reduce food waste at source using innovative ideas: (i) *Inventory rotation and labeling* - Retailers can adopt FIFO (first-in, first-out) systems and improve clarity on expiry labels to minimize spoilage.; (ii) *Surplus food donation* - Food retailers and restaurants should be encouraged to partner with food banks and community kitchens to divert edible surplus food away from landfill; (iii) *Cold chain reliability*: In areas prone to electricity interruptions, businesses may require support for generators or off-grid refrigeration to maintain food safety.

Globally, the trend across business is to harness energy, produce functional and efficient commodities e.g. to make hair, beauty, and skin products from food waste as well as creating clothing and textile from downstream food waste such as food packaging items. FLW alertness demands making resources available to impoverished people in ways aligned to cultural practices^22^ while teaching them contingency not to allow such bestowed value to go wasted, as Metz and Gaie posit^33^.

### Cultural framing: Ubuntu (Botho) and collective responsibility

The concept of Ubuntu (Botho)^3,17–23^, an African ethical philosophy emphasizing shared humanity and communal care, can support food sharing and waste reduction efforts. Ubuntu (Botho), often expressed as “I am because we are”, and as defined by Madondo^3^, promotes reciprocity and mutual responsibility, which aligns with resource-conscious behavior^19^. Framing food reuse and donation in a culturally grounded lens may enhance community acceptance and action.

## Conclusion

This study provides empirical insights into household food waste in Wallacedene, poverty stricken and largely informal settlement in Cape Town, contributing to the limited body of research on food waste in low-income urban areas of Sub-Saharan Africa. The findings reveal that 85% of households discard edible food, with vegetables, bread, and fruit being the most frequently wasted items. Food waste is driven by a combination of factors, including inadequate meal planning, limited food storage infrastructure, irregular access to electricity, and insufficient awareness of food preservation practices.

Although many residents expressed guilt or frustration over wasting food, structural and socioeconomic barriers often constrained their ability to reduce waste. This disconnect between intention and behavior reflects a broader pattern observed in global food waste literature and highlights the importance of addressing both behavioural and infrastructural dimensions.

From a policy perspective, the results underscore the need for multi-level interventions that include household education, improved municipal services, and targeted support for food redistribution networks. Culturally grounded strategies, such as those drawing on the principle of Ubuntu (Botho), may enhance community uptake, provided they are implemented sensitively and inclusively. Future research should aim to quantify food waste more precisely (e.g., through waste audits or kitchen diaries) and explore the effectiveness of specific interventions across informal settlements. Expanding the evidence base will be essential for developing scalable, context-appropriate solutions to food waste in rapidly urbanizing regions.

## Data Availability

Any reader who may wish to request the data from this study should contact the lead author: S’celo E. Madondo: Email: [scelomadondo@gmail.com](mailto: scelomadondo@gmail.com).

## References

[1] Giordano, C. et al. Household Food Waste Awareness in Relation to Motivations. Sustainability, 15(15), p.11582. (2023).

[2] Institution of Mechanical Engineers. Global food, waste want not. (accessed 9 March 2023).(2013). Available from: https://www.imeche.org/policy-and-press/reports/detail/global-food-waste-not-want-not (2013).

[3] Madondo, S. E. An assessment of household food wastage: A study of Wallacedene in Kraaifontein, Cape Town (Masters Dissertation, University of the Western Cape). (Accessed 10 October 2023). https://hdl.handle.net/10566/13027 (2022).

[4] Food and Agriculture Organization (FAO). Save food: Global initiative on food loss and waste reduction. Key facts on food loss and waste you should know! (accessed 23 October 2019). http://www.fao.org/food-loss-and-food-waste/en/ (2019).

[5] United Nations (UN). *Sustainable Development Goals (SDGs). Transforming our World: the 2030 Agenda for Sustainable Development* (United Nations, 2015).

[6] Buzby, J. C., Wells, H. F., & Aulakh, J. Food loss—Questions about the amount and causes still remain. Amber Waves: The Economics of Food, Farming, Natural Resources, and Rural America (5) (2014). http://ageconsearch.umn.edu

[7] Stancu, V., Haugaard, P. & Lähteenmäki, L. Determinants of consumer food waste behaviour: Two routes to food waste. *Appetite* **96**, 7–17. 10.1016/j.appet.2015.08.025 (2016). 26299713 10.1016/j.appet.2015.08.025

[8] Stuart, T. *Waste: Uncovering the Global Food Scandal* (WW Norton & Company, 2009).

[9] National Waste Management Strategy (NWMS). (accessed 17 June 2020). file:///C:/Users/LG/Desktop/Post%20Covid%20FW%20Documents/NVM-SNB-Report/2020nationalwaste_managementstrategy1.pdf (2020).

[10] Oelofse, S. H. Food waste in South Africa: Opportunities and challenges. (2015). http://researchspace.csir.co.za/dspace/bitstream/handle/10204/8748/Oelofse4_2015.pdf?sequence=1 [Accessed 22 August 2019].

[11] Cronjé, N., Van Der Merwe, I. & Müller, I. M. Household food waste: A case study in Kimberley, South Africa. *J. Consumer Sci.* **46** (2018).

[12] Shafiee-Jood, M. & Cai, X. Reducing food loss and waste to enhance food security and environmental sustainability. *Environ. Sci. Technol.* **50** (16), 8432–8443 (2016). 27428555 10.1021/acs.est.6b01993

[13] United Nations (UN). *United Nations Millennium Declaration (UNMD)* (United Nations, 2000).

[14] Gustavsson, J., Cederberg, C., Sonesson, U., Van Otterdijk, R. & Meybeck, A. *Global Food Losses and Food Waste* (FAO, 2011).

[15] Thyberg, K. L. & Tonjes, D. J. Drivers of food waste and their implications for sustainable policy development. *Resour. Conserv. Recycling* **106**, 110–123 (2016).

[16] Idso, C. D. *Estimates of Global Food Production in the Year 2050* (Center for the Study of Carbon Dioxide and Global Change, 2011).

[17] Fraser, W. J. & Ferreira, R. The pedagogy of Indigenous knowledge as a social construct. *Suid-Afrikaanse Tydskrif Vir Natuurwetenskap En Tegnologie*. **34** (1), 6 (2015).

[18] Metz, T. Ubuntu as a moral theory and human rights in South Africa. *Afr. Hum. Rights Law J.* **11** (2), 532–559 (2011). https://philarchive.org/archive/METUAA-2v1

[19] Metz, T. & Gaie, J. The African ethic of Ubuntu/Botho: Implications for research on morality. *J. Moral Educ.* **39**(3), 273–290. 10.1080/03057240.2010.497609 (2010).

[20] Moyo, B. H. & Thow, A. M. T. Fulfilling the right to food for South africa: justice, security, sovereignty and the politics of malnutrition. *World Nutr.* **11** (3), 112–152 (2020).

[21] Ngcoya, M. Ubuntu: toward an emancipatory cosmopolitanism? *Int. Political Sociol.* **9** (3), 248–262 (2015).

[22] Phasha, L. et al. Influence of cultural practices on food waste in South Africa—a review. *J. Ethnic Foods*. **7**, 1–13. 10.1186/s42779-020-00066-0 (2020).

[23] Xaba, T. R. Exploring the Principle of ubuntu in the South African Criminal Law System. University of Johannesburg (RSA). (2018). https://ujcontent.uj.ac.za/vital/access/manager/Index?site_name=Research%20Output

[24] Department of Environment, Forestry and Fisheries (DEFF) and Council for Scientific and Industrial Research (CSIR). Food waste prevention and management – A guideline for South Africa. Edition 1. DEFF and CSIR, Pretoria (accessed 19 August 2021). https://www.csir.co.za/sites/default/files/Documents/Food%20waste%20prevention_LANDSCAPE%28EDMS%29%20-%2005-02-2021.pdf (2021).

[25] Oelofse, S. H. & Nahman, A. Estimating the magnitude of food waste generated in South Africa. Waste Management & Research, 31(1), pp.80–86. The Journal of the International Solid Wastes and Public Cleansing Association, ISWA. 31. (2013). 10.1177/0734242X12457117 10.1177/0734242X12457117 22878934

[26] Stats, S. A. Towards measuring the extent of food security in South Africa: An examination of hunger and food adequacy. Statistics South Africa: Pretoria, South Africa. (accessed 15 December 2019). https://www.statssa.gov.za/?p=12135 (2019).

[27] Battersby, J. & Peyton, S. The geography of supermarkets in cape town: supermarket expansion and food access. *Urban Forum*. **25** (2), 153–164. 10.1007/s12132-014-9217-5 (2014).

[28] Oelofse, S. H., Muswema, A. & Ramukhwatho, F. Household food waste disposal in South africa: A case study of Johannesburg and Ekurhuleni. *South Afr. J. Sci*. **114** (5–6), 1–6. 10.17159/sajs.2018/20170284 (2018).

[29] Chauvin, N. D., Mulangu, F. & Porto, G. Food production and consumption trends in sub-Saharan Africa: Prospects for the transformation of the agricultural sector. UNDP Regional Bureau for Africa. New York, USA. City Population, 2011. Kraaifontein Census 2011. (accessed 6 May 2019). (2012). https://www.citypopulation.de/en/southafrica/cityofcapetown/199018__kraaifontein/

[30] Schott, A. B. S. & Andersson, T. Food waste minimization from a life-cycle perspective. *J. Environ. Manage.* **147**, 219–226. 10.1016/j.jenvman.2014.07.048 (2015). 25264296 10.1016/j.jenvman.2014.07.048

[31] Nahman, A. & De Lange, W. Costs of food waste along the value chain: evidence from South Africa. *Waste Manage.* **33** (11), 2493–2500 (2013). http://hdl.handle.net/10204/7124 10.1016/j.wasman.2013.07.012 23910243

[32] Russell, S. V., Young, C. W., Unsworth, K. L. & Robinson, C. Bringing habits and emotions into food waste behaviour. *Resour. Conserv. Recycling* **125**, 107–114. 10.1016/j.resconrec.2017.06.007 (2017).

[33] Katajajuuri, J. M., Silvennoinen, K., Hartikainen, H., Heikkilä, L. & Reinikainen, A. Food waste in the Finnish food chain. *J. Clean. Prod.* **73**, 322–329 (2014).

[34] Matinise, S. N., Roos, C., Oelofse, S. H. & Muswema, A. P. Implementing the waste hierarchy – Assessing the recycling potential of restaurant waste. Paper presented at Wastecon Conference, 16–18 October, Johannesburg, South Africa (2018).

[35] Hoballah, A. & Averous, S. Ensure sustainable consumption and production patterns. *UN Chron.* **51** (4), 28–29 (2015). https://www.un.org/en/chronicle/article/goal-12-ensuring-sustainable-consumption-and-production-patterns-essential-requirement-sustainable

[36] Parfitt, J., Barthel, M. & McNaughton, S. Food waste within food supply chains: Quantification and potential for change to 2050. *Philos. Trans. Royal Soc. B Biol. Sci.* **365**(1554), 3065–3081 (2010). 10.1098/rstb.2010.0126 PMC2935112 20713403

[37] Warehouse Food Banking (WFB). Promoting food security – Model for sourcing, collecting, and storing edible food to feed vulnerable communities by redistributing the food to vetted beneficiary organizations (2025). https://www.foodbanking.org/promoting-food-security/

[38] University of Stellenbosch (US). Wallacedene TRA: Community Risk Assessment. Research Alliance for Disaster and Risk Reduction (RADAR). (2015). https://www.radar.org.za/assets/files/Wallacedene%20TRA%20Community%20Risk%20Assessment%20Report.pdf [Accessed 28 May 2019].

[39] Ajzen, I. The theory of planned behavior. *Organ. Behav. Hum Decis. Process.* **50** (2), 179–211. 10.1016/0749-5978(91)90020-T (1991).

[40] Davis, J. Grootboom and Others v Oostenberg Municipality and Others (6826/99) [1999] ZAWCHC 1 (17 December 1999) SA: Western Cape High Court, Cape Town – Cape of Good Hope Provincial Division.

[41] Google Maps Wallacedene – geographical location. Cape Town, 7570. (2021). https://www.google.com/maps/place/Wallace+Dene,+Cape+Town,+7570/@-33.6920127,17.9398761,9z/data=!4m5!3m4!1s0x1dcc53c1d4e7b24b:0x3fdc371830b7993e!8m2!3d-33.8607445!4d18.7318822

[42] City Population Kraaifontein Census 2011. (accessed 6 November 2019) (2011). https://www.citypopulation.de/en/southafrica/cityofcapetown/199018__kraaifontein/.

[43] Stats, S. A. Census 2011: Statistical release – Revised P0301. Pretoria: Statistics South Africa. (accessed 10 April 2019). http://www.statssa.gov.za/ (2011).

[44] Nahman, A., De Lange, W., Oelofse, S. & Godfrey, L. The costs of household food waste in South Africa. *Waste Manag.* **32**(11), 2147–2153 (2012). 22608682 10.1016/j.wasman.2012.04.012

[45] Dladla, I., Machete, F. & Shale, K. A review of factors associated with indiscriminate dumping of waste in eleven African countries. *Afr. J. Sci. Technol. Innov. Dev.* **8** (5–6), 475–481. 10.1080/20421338.2016.1224613 (2016).

[46] Williams, H., Wikström, F., Otterbring, T., Löfgren, M. & Gustafsson, A. Reasons for household food waste with special attention to packaging. *J. Clean. Prod.* **24**, 141–148. 10.1016/j.jclepro.2011.11.044 (2012).

[47] Farr-Wharton, G., Foth, M. & Choi, J. H. Identifying factors that promote consumer behaviours causing expired domestic food waste. *J. Consumer Behav.* **13** (6), 393–402 (2014).

[48] Papargyropoulou, E., Lozano, R., Steinberger, J. K., Wright, N. & Ujang, Z. bin The food waste hierarchy as a framework for the management of food surplus and food waste. *J. Clean. Prod.* **76**, 106–115. 10.1016/j.jclepro.2014.04.020 (2014).

[49] Green Industries, S. A. Supporting the Circular Economy South Australia’s Waste Strategy 2020–2025. (accessed 7 July 2022). https://www.greenindustries.sa.gov.au/resources/sa-waste-strategy-2020-2025 (2020).

